# Final analysis of ArtemisR, a European real-world retrospective study of apalutamide for the treatment of patients with metastatic hormone-sensitive prostate cancer

**DOI:** 10.1186/s12885-025-14294-7

**Published:** 2025-07-01

**Authors:** Martin Boegemann, Mostefa Bennamoun, Louis Marie Dourthe, Juan Antonio Encarnacion, Axel Hegele, Eva Hellmis, Igor Latorzeff, Wolfgang Leicht, Julián Oñate-Celdrán, Antonio Rosino-Sánchez, Krystelle Hanna, Joana Lencart, Martin Lukac, Claudio A. Schioppa, Suzy Van Sanden, Gero Kramer

**Affiliations:** 1 Department of Urology, University Hospital Münster, Münster, Germany; 2 Oncology Department, Institut Mutualiste Montsouris, Paris, France; 3 Medical Oncology, Clinique Sainte-Anne, Strasbourg, France; 4 Radio-Oncology, University Hospital Virgen of Arrixaca, Murcia, Spain; 5 Urological Center Mittelhessen, Biedenkopf, Germany; 6 Department of Urology, University Hospital Marburg, Marburg, Germany; 7 Practice of Urology, Study Center, Urologicum Duisburg, Duisburg, Germany; 8 Clinique Pasteur, Groupe ORION, Toulouse, France; 9 Urology, Urology Hospital BarmHERZige Brüder Regensburg, Regensburg, Germany; 10 Hospital General Universitario Reina Sofía de Murcia, Murcia, Spain; 11 Urology, Hospital General Universitario Morales Meseguer, Murcia, Spain; 12 Janssen Pharmaceutica LLC, a, Johnson & Johnson Company, EMEA Medical Affairs , Dubai, United Arab Emirates; 13 Janssen Pharmaceutica LLC, a, Johnson & Johnson Company, EMEA Medical Affairs , Porto Salvo, Portugal; 14 Parexel International S.R.O. On Behalf of Janssen Pharmaceutica LLC, a Johnson & Johnson Company, Medical Affairs, Prague, Czech Republic; 15 Janssen Pharmaceutica LLC, Johnson & Johnson Company, EMEA Market Access Statistics, Analytics and Modelling, Beerse, Belgium; 16 Department of Urology, Comprehensive Cancer Center, Medical University of Vienna, Vienna, Austria

**Keywords:** Apalutamide, Metastatic hormone-sensitive prostate cancer, Prostate-specific antigen

## Abstract

**Background:**

We examined real-world outcomes of patients with metastatic hormone-sensitive prostate cancer (mHSPC) treated with apalutamide plus androgen deprivation therapy (ADT). The current study, reflecting real-life practice, included specific subpopulations not evaluated in the pivotal phase III TITAN study: patients diagnosed with novel imaging, patients with M1a disease only, and patients treated with concomitant radiotherapy.

**Methods:**

ArtemisR is the first European multi-country observational study to retrospectively collect data from medical records of patients with mHSPC treated with apalutamide plus ADT in routine clinical practice. Response rates of patients with 50% and 90% decrease in PSA level (PSA50 and PSA90) compared with baseline and undetectable PSA (uPSA, < 0.2 ng/mL) were reported. Time to PSA response, time to metastatic castration-resistant prostate cancer (mCRPC), proportion of apalutamide discontinuation, and survival at 12 months were also examined using the Kaplan–Meier method.

**Results:**

The analysis included 242 patients from Germany, France, Spain, and Austria; median age was 71 years. Median follow-up was 25.5 months from treatment initiation. Within 12 months of apalutamide initiation, 96% of patients achieved PSA50, 82% achieved PSA90, and 61% achieved uPSA. The median times to PSA50, PSA90, and uPSA were 1.08 months (95% confidence interval [CI]: 0.99–1.28), 1.94 months (95% CI: 1.54–2.27), and 3.48 months (95% CI: 2.92–5.68), respectively. At 12 months after treatment initiation, 94% of patients were alive, 91% had not progressed to mCRPC, and 81% remained on apalutamide plus ADT. Patients diagnosed with novel imaging, patients with M1a disease only, and patients treated with concomitant radiotherapy also showed deep and fast PSA responses (PSA90 and uPSA) with apalutamide plus ADT. Apalutamide-related adverse events (AEs) were reported in 90 patients (37%), and six patients (2.5%) discontinued apalutamide due to AEs. No new safety signals were detected.

**Conclusions:**

The ArtemisR European multi-centre study examined the efficacy and safety of apalutamide plus ADT for patients with mHSPC, further validating the deep and fast PSA response associated with this treatment regimen. These real-world outcomes were additionally observed in a more diverse patient population than that included in the pivotal TITAN study.

**Supplementary Information:**

The online version contains supplementary material available at 10.1186/s12885-025-14294-7.

## Introduction

Prostate cancer is the second most diagnosed cancer and fifth leading cause of cancer death in men worldwide [1]. The 5-year survival rate of patients with metastatic prostate cancer, such as metastatic hormone-sensitive prostate cancer (mHSPC; also known as metastatic castration-sensitive prostate cancer), is approximately 37% [2]. Efforts to improve these survival rates have led to expansion of the mHSPC treatment landscape over the past decades. International guidelines recommend that the standard of care for patients with mHSPC is androgen deprivation therapy (ADT) plus androgen receptor pathway inhibitors (ARPIs) with or without docetaxel [3–5], based on evidence from clinical trials demonstrating prolonged overall survival (OS), delayed disease progression, and maintained quality of life (QoL) with this doublet/triplet regimen compared with ADT alone or ADT plus docetaxel [6–10].

The ARPI apalutamide was approved by the European Medicines Agency (EMA) in 2019 for the treatment of adult men with non-metastatic castration-resistant prostate cancer, and is currently used in clinical practice [11, 12]. In 2020, the indication was expanded to use in adult men with mHSPC in combination with ADT [11] based on the results of the phase III TITAN study, which demonstrated that apalutamide combined with ADT significantly improved radiographic progression-free survival (PFS) and OS while maintaining health-related QoL compared with ADT alone in this population [13–15]. Post hoc landmark analyses of TITAN also showed that patients with mHSPC who were treated with apalutamide plus ADT had rapid, deep, and durable declines in prostate-specific antigen (PSA) levels versus baseline [16, 17]. Chowdhury et al. found that as early as 3 months after initiating treatment, 59% and 51% of the patients treated with apalutamide plus ADT achieved a PSA decline of ≥ 90% or to a level of ≤ 0.2 ng/mL compared with 13% and 18% of patients treated with ADT only, respectively, with similar declines continuing at 12 months [16]. Merseburger et al. showed even deeper PSA declines with 23% of patients treated with apalutamide plus ADT reaching ≤ 0.02 ng/mL at 3 months compared with just 5% of patients treated with ADT only [17]. Furthermore, deep declines in PSA were also associated with longer OS, radiographic PFS, time to PSA progression, and time to castration resistance [16, 17].

Beyond findings from clinical trials, there are limited real-world data documenting the PSA kinetics and tolerability of apalutamide for the treatment of patients with mHSPC in a European healthcare setting [18–20]. The aim of the ArtemisR study was, therefore, to further describe the use of apalutamide with ADT for the treatment of mHSPC in real-world clinical practice in four EU countries, including a broader and more diverse population than that evaluated in the TITAN study (i.e. including patients diagnosed with novel imaging, patients with M1a disease only, or patients treated with concomitant radiotherapy). Although the use of novel imaging techniques (such as prostate-specific membrane antigen positron emission tomography [PSMA-PET] and choline positron emission tomography [PET]/computerised tomography [CT]) has become more widespread in recent years [21, 22], the TITAN study only included patients diagnosed with conventional imaging. There is also growing interest in local treatment (such as radiotherapy) in combination with systemic therapy particularly for patients with low-volume mHSPC [23]; however, the TITAN study did not investigate the use of concomitant radiotherapy treatment with apalutamide. Finally, patients with M1a-only metastases were excluded from TITAN [13].

## Methods

### Study design and patients

ArtemisR was a retrospective, observational, multicentre study of patients with mHSPC treated with apalutamide plus ADT at specialist centres for urology or oncology across four European countries (Germany, France, Spain, and Austria). Eligible patients were adult men (aged 18 years and older) with histologically or cytologically confirmed diagnosis of mHSPC (based on physician’s discretion in accordance with current guidelines) who had started treatment with apalutamide plus ADT as part of clinical practice between the local date of apalutamide reimbursement and 30 August 2021. An informed consent form waiver or opt-out letter process, as applicable, has been followed based on country- and site-specific requirements. The study protocol was reviewed by Independent Ethics Committees/Institutional Review Boards at each participating institution and was conducted in accordance with applicable regulatory requirements.

### Data collection

The medical records of eligible patients were reviewed, and data were collected from the start of apalutamide treatment and covered at least 12 months of follow-up, unless the patient discontinued the study prior to this timepoint. Baseline data were documented, including patient age, PSA levels, clinically relevant comorbidities, Eastern Cooperative Oncology Group performance status, prostate cancer history, and prior anticancer therapy. Details of apalutamide treatment, time to discontinuation and reasons for discontinuation, concomitant treatment, relevant outcomes (including PSA levels), date of progression, and date of death were also recorded.

### Objectives and assessments

The primary objective of the study was to describe the real-world outcomes of the use of apalutamide for the treatment of male patients with mHSPC by assessing the rate of PSA response within different time windows (0–3 months, 0–6 months, 0–9 months and 0–12 months), time to PSA response, proportion of patients still on treatment at 12 months after treatment initiation, proportion of patients without metastatic castration-resistant prostate cancer (mCRPC) at 12 months, proportion of patients alive at 12 months, and safety.

Clinical response to apalutamide was evaluated based on data obtained as part of each patient’s standard of care, including PSA levels. PSA response was categorised according to the decrease in PSA levels versus baseline: 50% decrease (PSA50) and 90% decrease (PSA90). Patients with a baseline PSA level ≥ 0.2 ng/mL who experienced a decrease to < 0.2 ng/mL were considered to have an undetectable PSA (uPSA) response. Baseline PSA was defined as the closest PSA measurement within 90 days before and 3 days after initiation of apalutamide treatment. Further details of PSA response can be found in the supplement (Additional file 1 – Supplementary Methods). The proportion of patients alive at 12 months was also evaluated. Safety was evaluated based on documented incidence of adverse drug reactions (recorded from the first documented use of apalutamide until 30 days after the last documented use of apalutamide within the data collection period).

### Statistical analysis

This study was descriptive in nature and no hypotheses were tested. Baseline patient and disease characteristics were summarised using descriptive statistics. Time-to-event endpoints were analysed using the Kaplan–Meier method and median time-to-event variables and associated 95% confidence intervals (CIs) were reported. Further details of PSA measurements and of time-to-event endpoints can be found in the supplement (Additional file 1 – Supplementary methods).

Binary variables (PSA50, PSA90, and uPSA) were summarised by response rates (i.e. proportion of responders among patients with a record of at least one post-baseline PSA measurement) presented for specific time periods. Time-to-event and binary endpoints were also analysed for specific subgroups: patients diagnosed with novel imaging, patients with M1a disease only, and patients treated with concomitant radiotherapy.

A multivariable analysis of time to PSA response was also performed to investigate the simultaneous impact of several baseline characteristics on the outcome. The Cox proportional hazards model included characteristics considered to be clinically relevant: age, disease volume (high volume/low volume [based on the definition from the CHAARTED trial [24], with high volume defined per the information available in real-life practice: ≥ 4 bone lesions and/or visceral metastasis]), PSA at baseline, any visceral metastases at baseline, imaging method for diagnosis of mHSPC (conventional [CT, magnetic resonance imaging (MRI), bone scan] versus novel [whole-body MRI (WB-MRI), PSMA-PET, choline PET/CT, [^18^F]fluoro-deoxy-glucose ([^18^F]FDG) and other PET scans]), metastases subcategory at apalutamide treatment initiation (M1a, non-M1a), M0 at initial diagnosis (metachronous)/M1 at initial diagnosis (synchronous), Gleason score at initial diagnosis, concomitant radiotherapy during apalutamide treatment, and country. A *P* value was calculated from the model for each of these baseline characteristics, indicating overall statistical significance when *P* < 0.05.

## Results

### Patient population

In this retrospective analysis, data were collected for 242 patients from 24 urology/oncology centres across Germany, France, Spain, and Austria (Table 1) over a median duration of follow-up of 25.5 months. Patient median age was 71 (Q1–Q3: 66–77) years. At baseline, the median PSA level was 11.7 (Q1–Q3: 1.8–61.2) ng/mL. At treatment initiation, 128 patients (52.9%) were classified as having high-volume disease and 90 (37.2%) as having low-volume disease; classification for the remaining 24 (9.9%) was unknown. Among the subpopulations of interest, 28 patients (11.6%) had M1a disease (with non-regional lymph node disease only) at baseline, 80 of the 228 patients who had imaging at diagnosis (35.1%) were diagnosed with mHSPC using novel imaging, and 44 patients (18.2%) were treated with concomitant radiotherapy (a mix of radiotherapy to the prostate and/or to the metastases) during treatment with apalutamide. The complete list of patient characteristics at baseline is provided in Table 1.

**Table 1:** Patient characteristics

Characteristic	N = 242
Age, median (Q1–Q3), years	71 (66–77)
ECOG performance status at baseline, n (%)	
0	89 (36.8)
1	41 (16.9)
Unknown^a^	112 (46.3)
PSA, median (Q1–Q3), ng/mL	
At baseline^b^	11.7 (1.8–61.2)
Time from initial diagnosis to baseline, median (Q1–Q3), days^c^	113 (49–1080)
Disease subtype at baseline, n (%)	
High volume	128 (52.9)
Low volume	90 (37.2)
Unknown^a^	24 (9.9)
Metastases stage at initial diagnosis, n (%)	
M0 (metachronous)	59 (24.4)
M1 (synchronous)	156 (64.5)
Unknown^a^	27 (11.1)
Metastases stage subcategory at start of treatment, n (%)	
M1a	28 (11.6)
M1b	169 (69.8)
M1c	44 (18.2)
Unknown^a^	1 (0.4)
Number of metastases at start of treatment, n (%)	
≤ 5	86 (35.5)
> 5	141 (58.3)
Unknown^a^	15 (6.2)
Top 5 most common body system comorbidities, n (%)^d^	
Cardiovascular	141 (58.3)
Endocrine and metabolic	83 (34.3)
*Diabetes mellitus*	*46 (19.0)*
Genitourinary	37 (15.3)
Gastrointestinal	35 (14.5)
Musculoskeletal	26 (10.7)
Imaging method at time of mHSPC diagnosis, n (%)^e^	
Conventional imaging	147 (64.5)
Novel imaging	80 (35.1)
Unknown^a^	1 (0.4)
Prior docetaxel therapy, n (%)	5 (2.1)
Prior radiotherapy, n (%)	86 (35.5)
Prior radical prostatectomy, n (%)	51 (21.1)
Concomitant radiotherapy, n (%)	44 (18.2)
Palliative	7 (15.9)
Primary definitive therapy	10 (22.7)
Radiation for metastatic lesions	21 (47.7)
Unknown^a^	6 (13.6)
Country, n (%)	
Germany	73 (30.2)
France	71 (29.3)
Spain	85 (35.1)
Austria	13 (5.4)

*Abbreviations*: *ECOG* Eastern Cooperative Oncology Group, *mHSPC* metastatic hormone-sensitive prostate cancer, *PSA* prostate-specific antigen

^a^Data were reported as unknown where the relevant variables were not reported in the electronic records. ^b^N = 226. ^c^N = 237. ^d^Patients could have > 1 comorbidity, therefore the total can exceed 100%. ^e^N = 228 (two patients did not receive imaging and the imaging status was unknown for 12 patients)

### PSA response

**Fig. 1:**
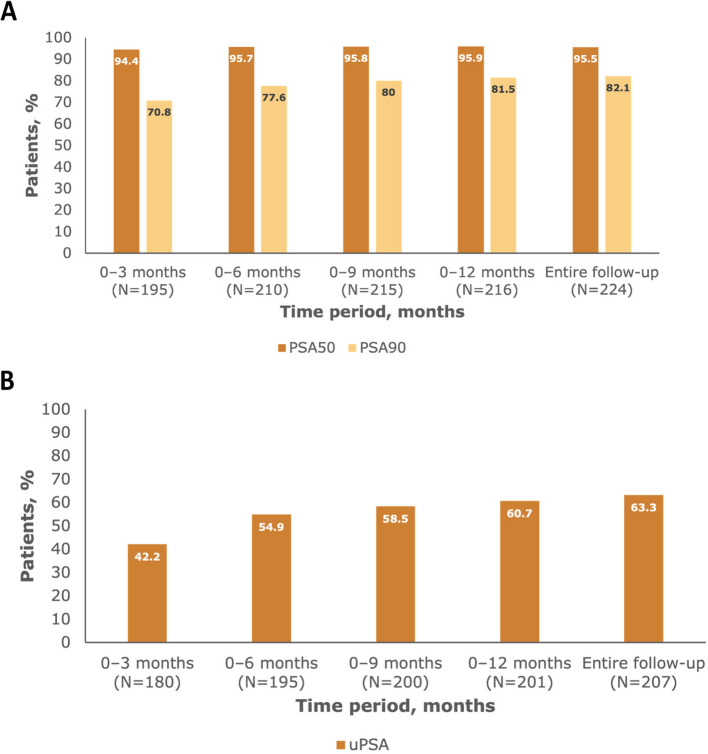
Patients achieving PSA50 and PSA90 responses (**A**) and uPSA response (**B**) for specific time periods. PSA response was categorised by decreases in PSA levels of 50% (PSA50) or 90% (PSA90) versus baseline. Patients with PSA levels ≥ 0.2 ng/mL at baseline who achieved PSA levels < 0.2 ng/mL were classified as having uPSA. N is the number of evaluable patients (with both baseline and ≥ 1 PSA measurement) per time period. Abbreviations: PSA, prostate-specific antigen; PSA50, 50% decrease in PSA level; PSA90, 90% decrease in PSA level; uPSA, undetectable PSA

**Fig. 2:**
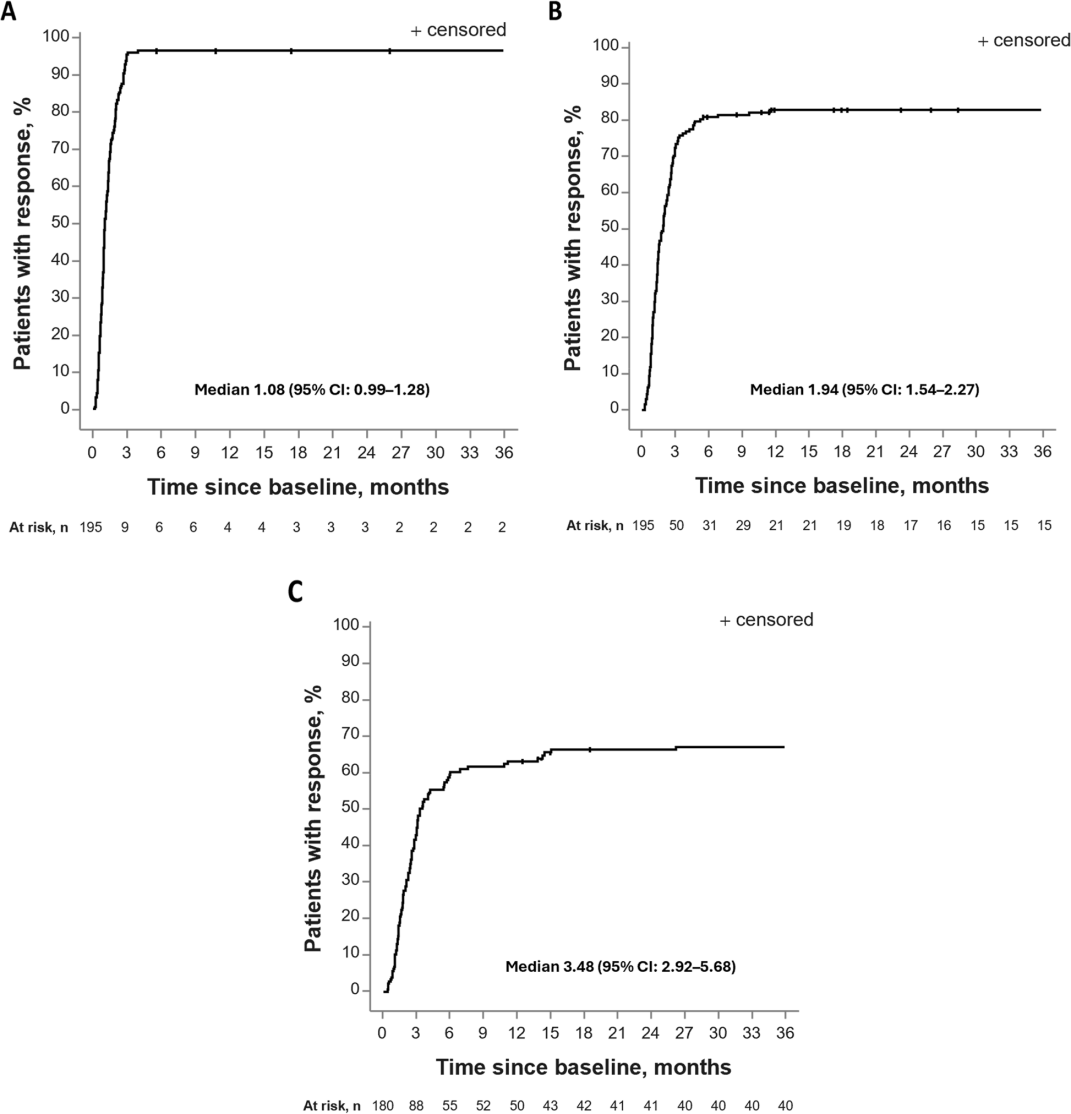
Time to PSA50 response (**A**), PSA90 response (**B**), and uPSA (**C**)^a^. Abbreviation: CI, confidence interval. ^a^ PSA response was defined as a decrease in PSA level from baseline of 50% (PSA50) or 90% (PSA90), or to < 0.2 ng/mL (uPSA), recorded during follow-up until the earliest between subsequent therapy and 3 months after treatment with apalutamide end. A response after switching to another therapy, or > 3 months after stopping apalutamide treatment was not counted

Within 3 months after initiation of apalutamide plus ADT treatment, 94.4% of evaluable patients achieved PSA50 and 70.8% achieved PSA90 (Fig. 1A); 42.2% of evaluable patients had achieved uPSA (Fig. 1B). Within 12 months, PSA50 response was achieved by 95.9% of patients and PSA90 by 81.5% (Fig. 1A); uPSA was achieved by 60.7% of evaluable patients (Fig. 1B). The median time to PSA50 was 1.08 months (95% CI: 0.99–1.28) (Fig. 2A), the median time to PSA90 response was 1.94 months (95% CI: 1.54–2.27) (Fig. 2B), and the median time to uPSA was 3.48 months (95% CI: 2.92–5.68) (Fig. 2C).

PSA response in specific subgroups, as well as multivariable analysis influencing time to PSA response, were investigated, specifically for PSA90 and uPSA due to clinical relevance.

#### PSA response in specific subgroups

In the subpopulation of patients in whom novel imaging techniques were used at diagnosis, 64.5% achieved PSA90 and 64.2% achieved uPSA within 3 months of apalutamide plus ADT treatment initiation (Table 2). Within 12 months, PSA90 was achieved by 82.6% of patients and uPSA by 81.7% (Table 2). The median times to PSA90 and uPSA were 2.04 months (95% CI: 1.48–2.66) and 1.81 months (95% CI: 1.48–2.37), respectively (Table 2).

**Table 2:** Best PSA response and median time to response stratified by metastatic stage subcategory, imaging method at diagnosis, and use of concomitant radiotherapy

Population	0–3 months		0–6 months		0–9 months		0–12 months		Median time to response, months	N^c^
	**%**^**a**^	**N**^**b**^	**%**^**a**^	**N**^**b**^	**%**^**a**^	**N**^**b**^	**%**^**a**^	**N**^**b**^	**(95% CI)**	
**All patients**										
PSA90	70.8	195	77.6	210	80.0	215	81.5	216	1.94 (1.54–2.27)	195
uPSA	42.2	180	54.9	195	58.5	200	60.7	201	3.48 (2.92–5.68)	180
**Novel imaging**										
PSA90	64.5	62	74.2	66	81.2	69	82.6	69	2.04 (1.48–2.66)	62
uPSA	64.2	53	75.4	57	80.0	60	81.7	60	1.81 (1.48–2.37)	53
**M1a**										
PSA90	50.0	20	66.7	24	76.9	26	81.5	27	2.99 (1.54–3.42)	20
uPSA	61.1	18	72.7	22	75.0	24	80.0	25	1.84 (1.54–3.09)	18
**Concomitant RT**										
PSA90	93.9	33	94.6	37	97.3	37	97.4	38	1.58 (0.92–1.74)	33
uPSA	54.5	33	67.6	37	70.3	37	73.7	38	2.60 (2.10–4.11)	33

*Abbreviations*: *CI* confidence interval, *PSA* prostate-specific antigen, *PSA50* 50% decrease in PSA level, *PSA90* 90% decrease in PSA level, *RT* radiotherapy, *uPSA* undetectable prostate-specific antigen

^a^Percentage of patients with response

^b^N is the number of evaluable patients (with both baseline and ≥ 1 PSA measurement) per time window

^c^N is the number of evaluable patients for Kaplan–Meier analysis (with both baseline and ≥ 1 PSA measurement in the first 3 months since treatment start)

Among the patients with M1a disease, 50.0% achieved PSA90 and 61.1% achieved uPSA within 3 months (Table 2). Within 12 months, PSA90 was achieved by 81.5% of patients and uPSA by 80.0% (Table 2). The median times to PSA90 and uPSA were 2.99 months (95% CI: 1.54–3.42) and 1.84 months (95% CI 1.54–3.09), respectively (Table 2).

Among the patients who received concomitant radiotherapy, 93.9% achieved PSA90 and 54.5% achieved uPSA within 3 months (Table 2). Within 12 months, PSA90 was achieved by 97.4% of patients and uPSA by 73.7% (Table 2). The median times to PSA90 and uPSA were 1.58 months (95% CI: 0.92–1.74) and 2.60 months (95% CI: 2.10–4.11), respectively (Table 2).

#### Multivariable analysis of factors influencing time to PSA response

**Fig. 3:**
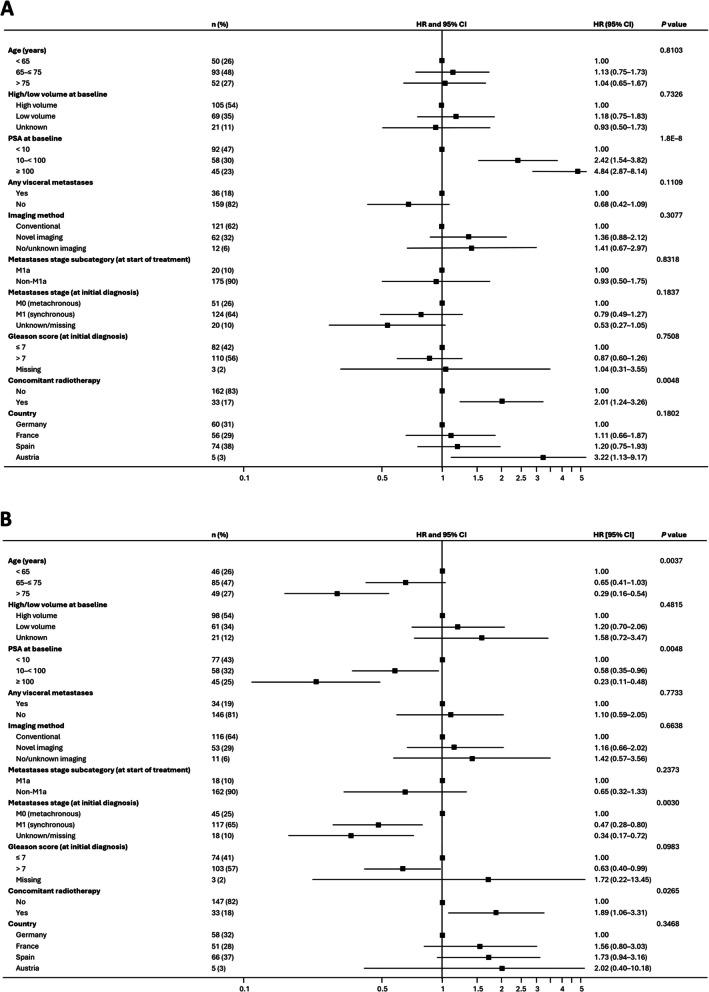
Multivariable analysis of time to PSA90 (**A**) and uPSA (**B**)^a.^ Abbreviations: CI, confidence interval; HR, hazard ratio; PSA, prostate-specific antigen; PSA90, 90% decrease in PSA level; uPSA, undetectable prostate-specific antigen. ^a^ HR > 1 indicates faster response, with respect to the reference category for each variable

The multivariable analysis of time to PSA response (PSA90 and uPSA) involved simultaneously adjusting for a range of baseline characteristics (Fig. 3). For PSA90, the characteristics with a statistically significant influence over the outcome were higher baseline PSA levels and receiving concomitant radiotherapy, both of which were associated with a faster response (Fig. 3A). For uPSA, statistically significant characteristics associated with a faster response were younger age (< 65 years), low baseline PSA levels (< 10 ng/mL), M0 disease stage at initial diagnosis (metachronous), and administration of concomitant radiotherapy (Fig. 3B). While a Gleason score of ≤ 7 at initial diagnosis was associated with quicker uPSA responses compared with a score > 7, the overall *P* value was not statistically significant, likely due to the considerable uncertainty surrounding the estimate for the three patients with missing Gleason scores.

### Treatment discontinuation

At 12 months after treatment initiation, 80.8% of patients remained on apalutamide treatment. Over the entire study follow-up period, 78 patients (32%) discontinued apalutamide treatment; of these, 32 went on to receive subsequent systematic anticancer treatment. Docetaxel (21 patients [65.7%]) was the most frequently received subsequent treatment, followed by enzalutamide (8 patients [25.0%]) and cabazitaxel (7 patients [21.9%]) (Table 3).

**Table 3:** Treatment received, patient disposition, and subsequent systematic therapies

Parameter	n (%)
Patients with dose reduction, n (%^a^)	17 (7)
Patients with dose interruption, n (%^a^)	8 (3.3)
Patients with treatment discontinuation, n (%^a^)	78 (32.2)
Reason for treatment discontinuation, n (%^b^)	
Disease progression	45 (57.7)
Adverse event	14 (17.9)
Death	11 (14.1)
Other	7 (9.0)
Missing	1 (1.3)
Subsequent systematic anticancer therapy received^c^, n (%)	32 (41.0)^d^
Docetaxel	21 (65.7)
Enzalutamide	8 (25.0)
Cabazitaxel	7 (21.9)
Abiraterone	5 (15.7)
Carboplatin	4 (12.5)
Etoposide	1 (3.1)
Lutetium	1 (3.1)
Mitoxantrone	1 (3.1)

^a^Proportion of patients from whole population (N = 242), or denominator is the whole population. ^b^Proportion of patients among the population of those who discontinued (N = 78). ^c^Patients may have used more than 1 anticancer therapy concurrently. ^d^Proportion of patients who received subsequent systematic therapy among those who discontinued apalutamide (N = 78)

### mCRPC and overall survival at 12 months

**Fig. 4:**
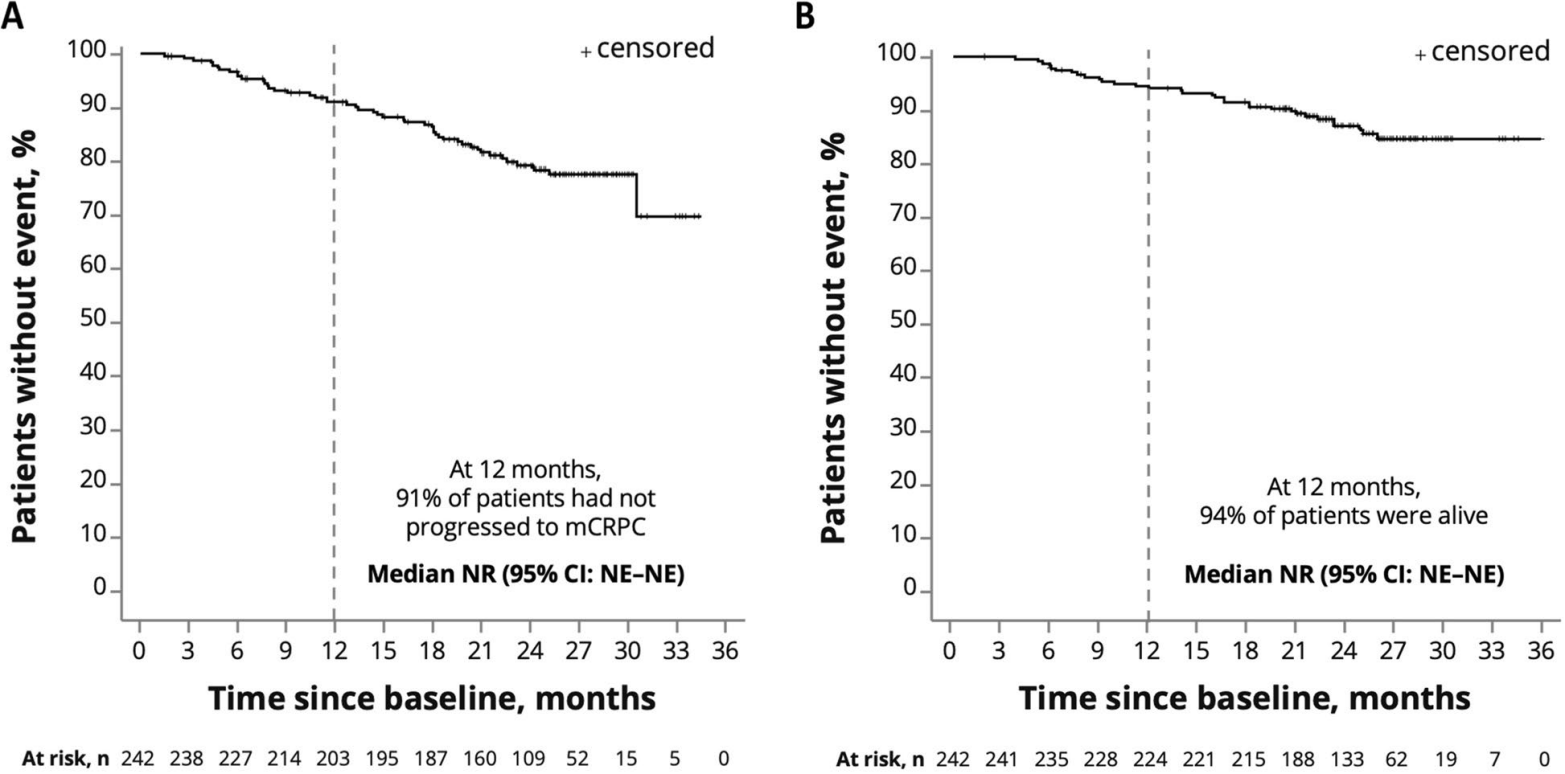
Time to mCRPC (**A**) and overall survival (**B**) among patients treated with apalutamide. Abbreviations: CI, confidence interval; mCRPC, metastatic castration-resistant prostate cancer; NE, not evaluable; NR, not reported

At 12 months after treatment initiation, 91.0% of patients had not progressed to mCRPC (Fig. 4A). The OS rate at 12 months was estimated to be 94.2% (Fig. 4B).

### Safety

A total of 90 patients (37.2%) reported treatment-related adverse events (TRAEs) (Table 4); 11 (4.5%) had Grade 3/4 TRAEs and 2 patients (0.8%) had serious TRAEs. TRAEs led to treatment discontinuation in six patients (2.5%). No fatal TRAEs were reported. The most common TRAEs were hot flushes (31 events), rash (23 events), and asthenia (18 events) (Additional file 1 – Table S1).

**Table 4:** Treatment-related adverse events among patients treated with apalutamide

n (%)	N = 242
Patients with TRAEs	90 (37.2)
Patients with Grade 3/4 TRAEs	11 (4.5)
Patients with serious TRAEs	2 (0.8)
Patients with fatal TRAEs	0
Patients with TRAEs leading to discontinuation	6 (2.5)
Patients with TRAEs leading to dose reduction	15 (6.2)

*Abbreviation*: *TRAE* treatment-related adverse event

## Discussion

ArtemisR is the first European, multi-country, observational study to retrospectively collect real-world data on treatment outcomes for patients treated with apalutamide for mHSPC. The findings from this study were consistent with results from the post hoc analysis of data from the TITAN study showing that apalutamide plus ADT treatment was associated with deep and fast declines in PSA levels [16].

The TITAN study demonstrated that 89%, 59%, and 51% of patients treated with apalutamide plus ADT achieved PSA50, PSA90, and PSA ≤ 0.2 ng/mL, respectively, as early as 3 months after treatment initiation, compared with 41%, 13%, and 18% of patients treated with ADT only [16]. Consistent results were observed in our study, and 94.4%, 70.8% and 42.2% of patients achieved PSA50, PSA90, and uPSA, respectively, within 3 months after initiation of apalutamide plus ADT treatment. PSA responses were also observed within 12 months of follow-up, with 95.9%, 81.5% and 60.7% of patients achieving PSA50, PSA90, and uPSA, respectively. The median times to PSA50, PSA90, and uPSA response in this study (1.08 months, 1.94 months, and 3.48 months, respectively) closely resembled the TITAN study results (particularly for PSA50 and PSA90 [1.0 and 1.9 months, respectively] with a time to response of 1.9 months for uPSA [16]). These findings were also comparable to that of a previous Spanish real-world study of apalutamide treatment in patients with mHSPC (median times to PSA50, PSA90, and PSA ≤ 0.2 ng/mL were 1 month, 1 month, and 3 months, respectively) [19]. In the current study, most patients were still alive at 12 months (94.2%) and had not yet progressed to mCRPC (91.0%). The rate of TRAEs observed in this study (37.2%) was consistent with the safety profile of apalutamide, despite 76.4% of the patients having comorbidities (including cardiovascular disease [58.3%] and diabetes mellitus [19%]), and no new safety signals were reported.

This study included analysis of patients with M1a disease, a subgroup that was not included in the TITAN study [13], also showing a deep and fast PSA decline in these patients.

Conventional imaging techniques such as MRI, CT, and bone scans have been used as standard in prostate cancer clinical trials for decades; however, in recent years, PET diagnostic methods, such as PSMA-PET and choline PET/CT, have demonstrated greater accuracy and sensitivity than conventional imaging allowing for earlier diagnosis of more advanced prostate cancer [21, 22]. Unlike the TITAN study, our investigation included all patients diagnosed with mHSPC in real-world clinical practice, including the subpopulation of patients diagnosed using novel imaging techniques (WB-MRI, PSMA-PET, choline PET/CT, [^18^F]FDG, and other PET scans). Our findings in this subpopulation provide further PSA outcome data for mHSPC patients diagnosed by novel imaging methods, which are increasingly used in real-world practice [19, 20].

Another advancement in mHSPC management is the recommended use of prostate radiotherapy for patients with newly diagnosed, low-volume disease [3, 5], largely based on the improved OS observed with radiotherapy plus standard of care compared with standard of care alone in this population in the STAMPEDE trial [25]. Metastasis-directed radiotherapy is another approach that is currently under clinical investigation and is already used in clinical practice [26]. Here, we show that in real-world practice, radiotherapy (including radiotherapy to the prostate and metastasis-directed radiotherapy) was combined with apalutamide in approximately one-fifth of patients, also showing a deep and fast PSA decline in these patients.

A multivariable analysis was conducted to explore the baseline characteristics that potentially impact the time to PSA response in patients treated with apalutamide. The results suggested that characteristics associated with improved time to uPSA included younger age, M0 disease stage at initial diagnosis (metachronous), Gleason score of ≤ 7 at diagnosis, and lower baseline PSA levels. It is interesting to note that it has been suggested that these characteristics are associated with better prognosis in prostate cancer [5, 27, 28]. A faster time to PSA90 response was associated with higher baseline PSA levels. In addition, administration of concomitant radiotherapy was found to be associated with both a faster time to uPSA and a faster time to PSA90 response. These findings align with results from a UK real-world study that showed improved PSA response when combining apalutamide with radiotherapy to the prostate [29]. Furthermore, the combination of apalutamide and stereotactic body radiotherapy is currently being investigated in a phase II trial in patients with oligometastatic mHSPC (NCT05717660) [30].

The results of this real-world analysis have important clinical implications. Apalutamide treatment resulted in a deep and fast PSA response in the majority of patients, confirming the findings of the TITAN study [13–15]. The importance of deep and fast PSA responses, as observed in the present study, has been highlighted previously in both clinical and real-world studies, showing correlation with improved outcomes such as OS and radiographic PFS [16, 18].

Limitations of this study included the descriptive nature of the analysis and the small sample sizes of each subgroup of interest (patients diagnosed with novel imaging, patients with M1a disease only, and patients treated with concomitant radiotherapy). As a result, the findings should be interpreted with caution and should be considered as hypothesis generating for further randomised trials. Further limitations included the shorter median follow-up time in comparison to the TITAN study (25.5 months versus 44.0 months), which prevented comparison of long-term oncological outcomes. Given the retrospective nature of this study, there was potential for bias due to factors such as patient selection, data missingness, and increased likelihood of underreporting of adverse events. Furthermore, as PSA measurements were taken per routine clinical practice, PSA data were not available for each 3-month window for all patients, unlike in the TITAN study. Although the multivariable analysis considered the simultaneous effect of many a priori clinically relevant baseline/disease characteristics, it cannot be excluded that other potentially relevant characteristics were not included in the model. Finally, real-world evidence may differ across countries due to variations in drug access, reimbursement and treatment protocols, which should be considered when interpreting our findings.

## Conclusion

The ArtemisR European multi-centre study examined the efficacy and safety of apalutamide plus ADT for patients with mHSPC, further validating the deep and fast PSA response associated with this treatment regimen. These real-world outcomes were additionally observed in a more diverse patient population than that included in the pivotal TITAN study.

## Supplementary Information

Additional file 1

## Data Availability

The data sharing policy of Janssen Pharmaceutical Companies of Johnson &; Johnson is available at https://www.janssen.com/clinical-trials/transparency. Although these data are not currently publicly available for sharing as they pertain to a non-interventional observational trial, requests for sharing can be sent to the Corresponding Author and will be evaluated on an individual basis.

## Abbreviations

ADT: Androgen deprivation therapy
ARPI: Androgen receptor pathway inhibitor
CI: Confidence interval
CT: Computerised tomography
EMA: European Medicines agency
[^18^F]FDG: [^18^F]-fluoro-deoxy-glucose
mCRPC: Metastatic castration-resistant prostate cancer
mHSPC: Metastatic hormone-sensitive prostate cancer
MRI: Magnetic resonance imaging
OS: Overall survival
PET: Positron emission tomography
PSA: Prostate-specific antigen
PSA50: 50% Decrease in PSA level
PSA90: 90% Decrease in PSA level
PSMA: Prostate-specific membrane antigen
QoL: Quality of life
TRAE: Treatment-related adverse event
uPSA: Undetectable PSA
WB-MRI: Whole-body MRI
