# Supplementary material for: Final analysis of ArtemisR, a European real-world retrospective study of apalutamide for the treatment of patients with metastatic hormone-sensitive prostate cancer
Source: BMC Cancer. 2025 Jul 1;25:1119. doi: 10.1186/s12885-025-14294-7 (PMC12211757; doi:10.1186/s12885-025-14294-7)
Supplement: Supplementary file 1 — Additional file 1 [file 12885_2025_14294_MOESM1_ESM.docx]

Supplementary appendix

## Supplementary methods

***Definition of PSA response***

- Baseline prostate-specific antigen (PSA) was defined as the closest PSA measurement within 90 days before and 3 days after the start of apalutamide treatment.
- For PSA response (PSA50, PSA90, uPSA) within a time interval, a patient was considered a PSA responder if they had a PSA decrease from baseline of 50% (PSA50), 90% (PSA90) or had a PSA value < 0.2 ng/ml (uPSA) within that time window. However, a response after switching to another therapy or more than 3 months after stopping apalutamide treatment was not counted as a response.
- Patients included in the analysis had to have a PSA measurement at baseline and at least 1 PSA measurement after baseline. For evaluation of uPSA response, only patients who had baseline PSA ≥ 0.2 ng/mL were included.

***Definition of time-to-event endpoints***

- Time-to-event was defined as the time from the start of apalutamide treatment until the date of the PSA measurement at which the endpoint was reached, with the following exceptions
  - If there was no PSA measurement in a 3-month window (0–3 months, 3–6 months, 6–9 months, etc.) then patients were either censored at the last measurement before that window (if the patient did not die and did not switch therapy in that window) or were censored at the last overall observed follow-up time (if the patient died or switched to subsequent therapy within 3 months of the last PSA measurement before the window)
  - If there was no such window without PSA measurement and the patient died or switched to subsequent therapy without experiencing an event first, they were censored at the last overall observed follow-up time.
- Patients who did not discontinue treatment were censored at the date of the last observation.

## Supplementary table

**Table S1** Most frequently occurring treatment-related adverse events by System Organ Class (occurring in ≥ 4 cases per System Organ Class)

| **System Organ Classes**  TRAEs: High-Level Terms | **Events, n (%)** | | | |  |
| --- | --- | --- | --- | --- | --- |
|  | **Grade 1** | **Grade 2** | **Grade 3** | **Grade 4** | **Total** |
| **Skin and subcutaneous tissue disorders**  Rashes, eruptions, and exanthems  Dermatitis and eczema  Apocrine and eccrine gland disorders  Pruritus  Dermal and epidermal conditions  Exfoliative conditions  Erythemas  Acnes | **22 (9.1)**  13 (5.4)  2 (0.8)  3 (1.2)  1 (0.4)  2 (0.8)  –  1 (0.4)  – | **8 (3.3)**  4 (1.7)  –  –  2 (0.8)  1 (0.4)  –  –  1 (0.4) | **8 (3.3)**  7 (2.9)  1 (0.4)  –  –  –  –  –  – | **1 (0.4)**  –  –  –  –  –  1 (0.4)  –  – | **39 (16.1)**  24 (9.9)  3 (1.2)  3 (1.2)  3 (1.2)  3 (1.2)  1 (0.4)  1 (0.4)  1 (0.4) |
| **Vascular disorders**  Peripheral vascular disorders  Vascular hypertensive disorders | **26 (10.7)**  26 (10.7)  – | **7 (2.9)**  6 (2.5)  1 (0.4) | –  –  – | –  –  – | **33 (13.6)**  32 (13.2)  1 (0.4) |
| **General disorders and administration site conditions**  Asthenic conditions  Oedema  General signs and symptoms | **26 (10.7)**  21 (8.7)  5 (2.1)  – | **5 (2.1)**  3 (1.2)  1 (0.4)  1 (0.4) | **1 (0.4)**  1 (0.4)  –  – | –  –  –  – | **32 (13.2)**  25 (10.3)  6 (2.5)  1 (0.4) |
| **Endocrine disorders**  Thyroid hypofunction disorders | **4 (1.7)**  4 (1.7) | **9 (3.7)**  9 (3.7) | –  – | –  – | **13 (5.4)**  13 (5.4) |
| **Musculoskeletal and connective tissue disorders**  Joint-related signs and symptoms  Bone-related signs and symptoms  Muscle weakness conditions  Muscle-related signs and symptoms | **9 (3.7)**  6 (2.5)  1 (0.4)  1 (0.4)  1 (0.4) | **1 (0.4)**  1 (0.4)  –  –  – | –  –  –  –  – | –  –  –  –  – | **10 (4.1)**  7 (2.9)  1 (0.4)  1 (0.4)  1 (0.4) |
| **Gastrointestinal disorders**  Nausea and vomiting symptoms  Gastrointestinal atonic and hypomotility disorders  Diarrhoea (excluding infective)  Gastrointestinal and abdominal pains (excluding oral and throat) | **2 (0.8)**  –  1 (0.4)  –  1 (0.4) | **2 (0.8)**  1 (0.4)  –  1 (0.4)  – | –  –  –  –  – | –  –  –  –  – | **4 (1.7)**  1 (0.4)  1 (0.4)  1 (0.4)  1 (0.4) |
| **Psychiatric disorders**  Confusion and disorientation  Anxiety symptoms  Disturbances in initiating and maintaining sleep  Sexual desire disorders | **3 (1.2)**  –  1 (0.4)  1 (0.4)  1 (0.4) | –  –  –  –  – | –  –  –  –  – | **1 (0.4)**  1 (0.4)  –  –  – | **4 (1.7)**  1 (0.4)  1 (0.4)  1 (0.4)  1 (0.4) |

Sum of events across High Level Terms or System Organ Classes does not represent the total number of patients with adverse events as multiple events per patient were possible. If multiple events per patient were reported for the same term, the highest-grade event was counted.

MedDRA dictionary (version 26.1) was used to group TRAEs in High Level Terms and System Organ Classes.

Abbreviations: MedDRA, Medical Dictionary for Regulatory Activities; TRAE, treatment-related adverse event
